# Craniofacial parameters and obstructive sleep apnea in newly diagnosed acromegaly

**DOI:** 10.3389/fendo.2025.1632944

**Published:** 2025-07-28

**Authors:** Magdalena Pilarska, Karolina Dżaman, Dawid Szczepański, Tomasz Węgrzecki

**Affiliations:** ^1^ Department of Otolaryngology, The Medical Centre of Postgraduate Education, Warsaw, Poland; ^2^ Department of Imaging Diagnostics, Bielanski Hospital, Warsaw, Poland

**Keywords:** acromegaly, obstructive sleep apnea, growth hormone, OSA, craniofacial deformities observational, cross-sectional study

## Abstract

**Introduction:**

Acromegaly is a rare condition caused by excess growth hormone after skeletal maturity, leading to abnormal soft tissue and bone growth. These changes raise the risk of obstructive sleep apnea (OSA) due to craniofacial abnormalities.

**Objective:**

The study aims to determine the correlation between the occurrence and severity of OSA and craniofacial anthropometric parameters in patients with newly diagnosed acromegaly.

**Study design:**

Observational, cross-sectional study.

**Setting:**

Single-center study involving patients diagnosed with acromegaly.

**Methods:**

The research included 30 patients ranging from 25 to 81 years old (mean age 48) who were diagnosed with acromegaly. The diagnosis of OSA relied on polygraphy with the SOMNO check micro device. MRI provided the necessary craniofacial and upper airway measurements. Each patient received an ear, nose, and throat examination followed by a fiberoptic evaluation of the upper airway.

**Results:**

OSA was diagnosed in 76.67% of patients. The analysis revealed that moderate to severe OSA affected 46% of patients, while women developed the condition at twice the rate of men. The research established a statistically relevant link between the severity of OSA and tongue base hypertrophy. The study failed to detect meaningful relationships concerning OSA severity and palatine uvula hypertrophy on MRI and between OSA severity and palatine tonsil size and middle pharyngeal airway width.

**Conclusion:**

Our study found a high OSA prevalence (76.67%) in newly diagnosed acromegaly patients and a significant association between tongue base hypertrophy (FTP scale) and OSA severity (p < 0.001), while other anatomical parameters showed no significant correlation with AHI. The high prevalence of OSA in patients with newly diagnosed acromegaly highlights the importance of including sleep apnea screening in the initial diagnostic workup.

## 1 Introduction

Acromegaly occurs as a result of the excessive production of growth hormone (GH) after the completion of skeletal growth. The majority of patients (99%) present with autonomous GH secretion by a pituitary adenoma. The condition can also arise from ectopic growth hormone-releasing hormone (GHRH) secretion by neuroendocrine tumors located in the pancreas and thymus or bronchial carcinoids (1, 2).

GH triggers the liver and peripheral tissues to generate somatomedins, particularly somatomedin C, which is also known as insulin-like growth factor 1 (IGF-1). GH stimulates cellular growth through IGF-1, which drives the excessive expansion of both bones and soft tissues. GH is responsible for tissue swelling along with sodium retention through three mechanisms that include stimulating glycosaminoglycan production, elastic connective tissue degradation, and epithelial sodium channel activation (3).

Elevated GH concentrations during adulthood induce the excessive enlargement of bones together with their surrounding soft tissues. The condition results in enlarged extremities as well as an increased size of the nose, ears, jaw and other body components. Other common features may include joint pain, facial changes, headaches, visual disturbances and comorbidities such as hypertension and diabetes. The combination of enlarged pharyngeal tissues with acromegalic craniofacial modifications raises the likelihood of developing obstructive sleep apnea (OSA). Research indicates that OSA develops in acromegalic patients through multiple factors, including craniofacial deformities (4–6) as well as hypertrophy of the soft palate, uvula and tongue (7, 8).

During sleep, the walls of the upper airway collapse to produce obstructive apnea, which results in a reduction of airway lumen size (9). The condition presents either as complete airflow interruption for 10 seconds or as significant airflow reduction reaching 90% below normal, while inspiratory effort remains steady or increases. Research shows that OSA affects between 20–80% of newly diagnosed acromegaly patients as well as 21–58% of patients with controlled disease (10, 11).

OSA increases cardiovascular disease risk, which, together with acromegaly-related complications like hypertension, cardiomyopathy, valvular defects and heart failure, leads to higher mortality rates in acromegaly patients (12, 13). It also increases some psychopathological changes related to acromegaly, with particular emphasis to anxiety and depression (14) and cancer risk (11, 15).

### 1.1 Aim of the study

The study aims to determine the correlation between the occurrence and severity of sleep apnea and craniofacial anthropometric parameters in patients with newly diagnosed acromegaly.

## 2 Materials and methods

### 2.1 Materials

This observational, cross-sectional study included patients with newly diagnosed acromegaly who presented with elevated GH and IGF-1 levels and had a pituitary macroadenoma confirmed on MRI. The research study included 30 participants between 25 and 81 years old, with a mean age of 48. The research group contained 40% women (n=12; mean age 58.75 years) together with 60% men (n=18; mean age 43 years).

All patients received BMI assessments, which showed an average BMI of 28.39 kg/m². The study revealed that 60% of participants (n=18) were overweight (BMI 25–29.99 kg/m²) and 23% (n=7) were obese (BMI >30 kg/m²). Evaluations were conducted at the Department of Otolaryngology and the Department of Endocrinology at Bielanski Hospital in Warsaw.

### 2.2 Methods

#### 2.2.1 Assessment of sleep-disordered breathing

The screening of sleep-disordered breathing episodes was performed using cardiorespiratory polygraphy with the SOMNO check micro device (Lowenstein Medical). The device recorded four physiological sleep parameters, including respiratory effort (via thoracic and abdominal movement), blood oxygen saturation, and airflow through the upper airway. The study was conducted according to the Level III diagnostic criteria of the AASM. The analysis focused on the AHI, which is the number of apnea and hypopnea events per hour of sleep. The following thresholds were applied:

- AHI 1–4: within normal range
- AHI 5–15: mild OSA
- AHI 16–30: moderate OSA
- AHI >30: severe OSA

#### 2.2.2 Anthropometric assessment of the craniofacial region and upper airways in MRI

**Figure 1:**
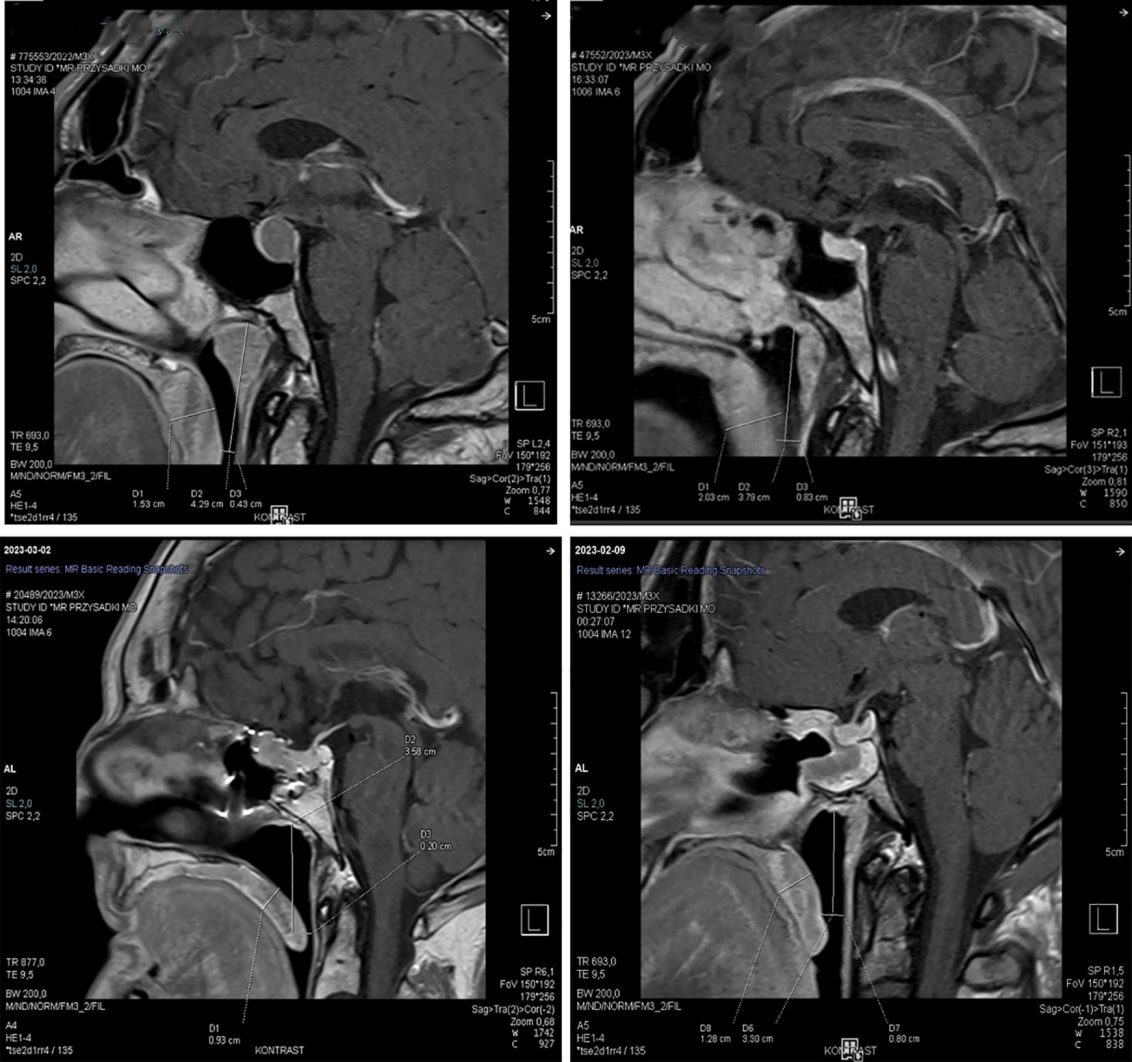
Example of pharyngeal structure measurements: measurement of uvular thickness and middle pharyngeal airway width. Measurement level - made in the sagittal plane, 1 cm above the inferior border of the uvula.

Craniofacial MRI was used to obtain measurements of the palatine uvula thickness and the width of the middle pharyngeal airway. Measurements were made in the sagittal plane, 1 cm above the inferior border of the uvula. The width of the middle pharynx was determined by measuring the distance between the posterior pharyngeal wall and the posterior surface of the uvula (**Figure 1**).

#### 2.2.3 ENT examination

A single ENT specialist conducted the otolaryngological assessment to evaluate craniofacial morphological variations. The assessment of nasal patency occurred through anterior rhinoscopy and nasal endoscopy. Two independent Friedman grading systems evaluated the oropharyngeal region to assess the tongue shape, palatine tonsils, and soft palate structures.

The four-grade FTP scale served to evaluate tongue placement in relation to tonsils and palatal arches during open mouth and resting tongue position on the floor of the mouth. The following classification was used:

Grade I – The entire uvula, together with tonsils and palatal arches, was clearly visible to the examiner.
Grade II – The majority of the uvula remained visible, but the tonsils and palatal arches appeared partially hidden.
Grade III – A small portion of either the soft palate or its distal end remained discernible to the examiner.
Grade IV – The observer could only see the hard palate.

The second Friedman scale evaluated the size of palatine tonsils in relation to the palatal arches:

Grade I – Absence of tonsils.
Grade II – Tonsils remained entirely within the boundaries of the palatal arches.
Grade III – The tonsils reached past the boundaries of the palatal arches.
Grade IV – The tonsils touched at the midline point.

The assessment of epiglottis and laryngeal structures took place through nasofiberoscopic visualization of the upper airway.

The study was conducted in accordance with the Declaration of Helsinki, and approved by the Bioethical Committee of the Centre of Postgraduate Medical Education (approval code: KD 435/2023, approval date: 13 December 2023).

#### 2.2.4 Statistical analysis

We set the significance level at α = 0.05, in accordance with established norms in biomedical research. To assess the normality of the distribution of continuous numerical variables, we applied the Shapiro-Wilk test. For variables that followed a normal distribution, descriptive statistics were reported as the mean (*M*) and standard deviation (*SD*). For non-normally distributed variables, we presented the median (*Mdn*) along with the first (*Q1*) and third quartiles (*Q3*). Categorical variables were summarized using frequencies and percentages, providing a clear depiction of their distribution within the cohort.

Comparative analyses were performed between two distinct patient groups, stratified based on the presence or absence of Obstructive Sleep Apnea (OSA). For continuous variables, the Wilcoxon rank-sum test was employed to detect differences, as it is a non-parametric test that does not assume normality of the data. In cases where the data followed a normal distribution, the Welch t-test was used. Effect sizes were calculated to quantify the magnitude of differences; Cohen’s *d* was reported for parametric tests, while the point-biserial correlation (*r_point-biserial_*) was used for non-parametric tests.

Due to the low expected frequencies of some categorical variables, statistical significance for these variables was determined using Fisher’s exact test, which is appropriate for small sample sizes or sparse data.

For assessing the relationship between two continuous variables, where at least one variable deviated from normality, we used Spearman’s rank correlation (*rho*). The p-value was approximated from the t-test statistic to determine the statistical significance of the correlation.

## 3 Results

### 3.1 Polygraphy findings

**Figure 2:**
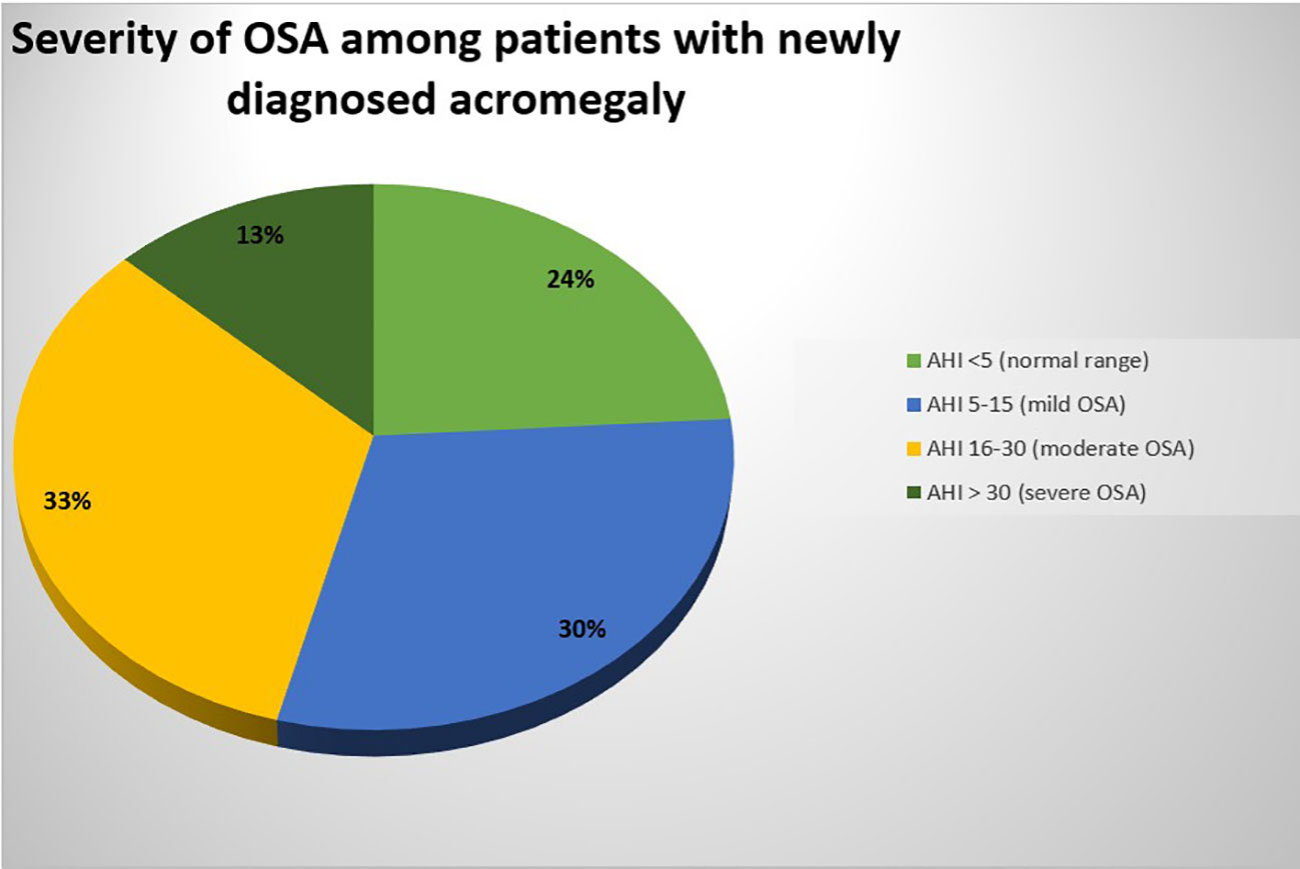
Severity of OSA among patients with newly diagnosed acromegaly.

Cardiorespiratory polygraphy tests revealed OSA in 76.67% of patients newly diagnosed with acromegaly. The sleep study showed that 30% of patients had mild OSA, 33% had moderate OSA, and 13% had severe OSA (**Figure 2**). The study found no meaningful statistical difference in OSA occurrence between male and female patients (p=1.000). The diagnosis of OSA occurred in 75% of women (n=9) and 77.78% of men (n=14). However, a significant sex-related difference in OSA severity was observed. The results showed that men had significantly more cases of mild OSA than women (p=0.035), with 44.44% of men (n=8) versus 8.33% of women (n=1).

The results indicated a possible higher occurrence of moderate and severe OSA among female patients, but this finding failed to achieve statistical significance. The analysis revealed that moderate OSA affected 50% of women, but only 22.22% of men, while severe OSA affected 16.67% of women, but only 11.11% of men (p=0.141).

An additional analysis was performed to determine the connection between IGF-1 values and the degree of OSA. Patients who had AHI <5 showed IGF-1 levels averaging 619.57 ng/ml, while those with AHI >5 showed levels averaging 605.2 ng/ml. The measured difference between these two groups was not statistically significant.

### 3.2 Otolaryngological examination results

The ENT examination revealed tongue hypertrophy in 73% of patients. The position of the tongue was evaluated through the FTP scale. The FTP grading system placed 53% of patients into grade III and 24% into grade II.

The AHI was further analyzed in relation to the FTP grading system. The research demonstrated that patients with higher FTP scores showed a statistically significant link to OSA development (p<0.001). The analysis between FTP classification and BMI failed to demonstrate any meaningful statistical relationship (p=0.179).

The Friedman Tonsil Grading Scale served to evaluate the dimensions of palatine tonsils. The palatal arches revealed tonsillar extension in 35% of patients: grade III classification was assigned to 32% of patients, and grade IV to 3%. The remaining 65% of cases showed tonsils that did not surpass the arches and received grade II classification.

### 3.3 Results of craniofacial and upper airway anthropometric assessment on MRI

The assessment of uvular thickness and middle pharyngeal airway width by MRI was conducted in 24 out of 30 patients (**Table 1**). The remaining patients received only report summaries from their MRI evaluations because the diagnostic imaging data were unavailable.

**Table 1:** Summary of hormonal levels, polygraphy data, and craniofacial measurements.

No.	Age (years)	Sex (M/F)	BMI	IGF-1 (ng/ml)	GH (ug/l)	AHI index	Uvular Thickness (mm)	MPAW (mm)	FTP
1.	59	M	43.65	109.6	<0.05	52.8	—	—	3
2.	57	F	15.99	333.4	3.61	20.9	12.8	6.1	2
3.	76	M	29.04	789.2	8.35	22.8	—	—	3
4.	62	F	26.56	677.2	3.49	28.4	12.4	2.9	4
5.	62	M	26.06	271.6	0.65	24.6	12.8	2.1	3
6.	41	M	24.22	665.9	4.51	4.5	11.2	3.5	1
7.	81	F	30.48	577.8	1.17	18.3	14.0	3.1	3
8.	45	M	28.09	640.0	4.69	14.7	—	—	3
9.	43	F	21.87	400.3	1.83	1.2	10.7	2.4	1
10.	69	F	26.4	749.0	4.90	50.9	13.1	1.8	4
11.	44	M	31.96	940.8	13.9	9.3	13.0	5.5	3
12.	41	M	29.67	323.0	0.13	1.3	14.9	4.6	1
13.	42	M	31.83	595.1	4.36	10.0	—	—	3
14.	58	M	26.83	843.0	17.5	21.9	17.2	4.1	3
15.	31	M	28.68	431.4	1.10	5.2	14.3	6.9	2
16.	48	F	29.49	890.1	7.75	5.1	11.1	2.5	2
17.	44	M	26.25	416.0	2.93	11.0	15.5	0	3
18.	44	M	29.88	1047.0	11.7	22.3	16.5	6.0	3
19.	39	M	32.72	553.2	2.06	7.8	15.8	8.0	2
20.	42	F	25.28	479.9	15.5	3.3	10.5	4.6	1
21.	51	F	25.14	468.8	11.9	16.0	14.5	3.0	3
22.	58	F	25.71	713.1	>80	21.8	—	—	3
23.	66	F	23.15	360.1	2.95	23.6	11.7	5.3	2
24.	26	M	16.01	665.5	32.0	2.1	13	8	2
25.	30	M	28.73	983.2	>80	4.0	15.4	4.3	2
26.	53	M	30.86	766.0	27.60	5.5	—	—	3
27.	25	F	29.07	819.2	18.60	1.3	10.2	5.3	1
28.	48	M	26.59	641.1	25.50	9.9	12.4	2.8	3
29.	53	M	30.03	689.0	13.20	38.6	22.5	11.7	3
30.	75	F	29.33	418	12.5	36	10.3	1.6	3

MPAW, Middle Pharyngeal Airway Width; FTP, Friedmann Tongue Position.

Reference ranges: GH 0.1–1.23 µg/L. IGF-1 65–222 ng/mL. AHI <5/h. BMI 18–25.

The mean uvular thickness of patients with acromegaly measured 13.00 mm (IQR: 11.58–15.03 mm). The uvular measurements of patients with OSA showed a higher median value at 13.10 mm (IQR: 12.40–15.50 mm) than those without OSA at 11.20 mm (IQR: 10.60–13.95 mm). The lack of statistical significance (p = 0.135) did not support the clinical observation of more pronounced uvular hypertrophy in patients with confirmed OSA.

The researchers evaluated uvular thickness in relation to IGF-1 concentration measurements. The Spearman’s rank correlation analysis showed a weak positive relationship between these variables, which failed to reach statistical significance (p = 0.354).

The study group displayed a middle pharyngeal airway width (MPAW) range of 1.6 mm to 11.7 mm with an average measurement of 4.42 mm. The average airway width of patients with diagnosed OSA measured 4.32 mm (SD = 2.86), whereas patients without OSA showed an average of 4.67 mm (SD = 1.74). The difference was not statistically significant. **Table 1** contains a summary of hormonal levels, polygraphy data, and craniofacial measurements.

## 4 Discussion

Acromegaly is a rare disorder due to the excessive secretion of GH from the pituitary gland, which results in high levels of IGF-1. Most often, this hormonal imbalance is caused by a pituitary adenoma (16). According to epidemiological data. the incidence of acromegaly varies from 2 to 11 cases per million people per year (17, 18). The disease seems to affect both sexes equally, which follows the sex distribution in our study group. The pattern of OSA in patients with acromegaly is different from that observed in the general population, where OSA is approximately twice as common in men. In contrast, our results show that women with acromegaly are more frequently affected by moderate to severe forms of OSA than men.

Acromegaly is usually diagnosed in the fourth or fifth decade of life, but the disease is believed to start about ten years earlier. In our cohort, more than half of the patients were between 30 and 50 years of age, and another 30% were between 50 and 70 years of age.

Hypertrophy of soft tissues in the upper airway, including macroglossia, can cause airway narrowing and collapse during sleep and is a frequent complication in acromegalic patients. This association has been known for more than a century. Chappel and Booth described abnormal ENT findings in 1896 (19).

Polysomnography remains the diagnostic gold standard for OSA (16, 18, 20), but cardiorespiratory polygraphy is a recognized and more accessible alternative in clinical practice.

Studies present conflicting data about how often OSA occurs among people with active acromegaly. The reported frequency of OSA diagnosis differs between current patients and those with controlled disease (7, 11). Our research demonstrated that OSA affected 76% of patients who received their acromegaly diagnosis for the first time. 66% of patients exhibited either moderate or severe sleep-disordered breathing according to their diagnostic results.

The research did not establish a statistical relationship between IGF-1 values and OSA severity. but other studies have demonstrated decreased apneic episode numbers during treatment. When sleep studies were conducted after one and three months of treatment, none of the patients experienced complete resolution of their apneas (21).

OSA in acromegalic patients is caused by pharyngeal and oral cavity hypertrophy, and craniofacial abnormalities. The obstruction occurs due to pharyngeal wall collapse, which happens at various points (22). Each patient with acromegaly displays distinct anatomical features of their upper airway passage. The research has not identified a primary site of airway obstruction responsible for apnea in this patient group. Endoscopic examinations performed under pharmacologically induced sleep have demonstrated that upper airway collapse may involve several mechanisms, including retraction of the tongue base, collapse of the lateral pharyngeal walls at both the mid- and lower pharyngeal levels, supraglottic collapse and posterior displacement of the soft palate and uvula toward the pharyngeal wall (22).

Patients with acromegaly show hypertrophy of both the soft palate and uvula, along with elongation of the uvula in many cases. Some researchers have suggested that these anatomical findings visible through MRI may lead to reduced dimensions of the middle pharyngeal airway, particularly in patients who have moderate to severe OSA (11). Our study did not demonstrate any statistically relevant connection between uvular thickness measurements and AHI results (p = 0.135) or between pharyngeal width measurements and AHI results (p = 0.716). The observed pattern showed that OSA patients had uvula measurements ranging from 12.4 to 15.5 mm while patients without OSA had measurements from 11.6 to 13.95 mm. The lack of statistical significance may have been avoided with a larger sample size. Palatine tonsils were responsible for the airway constriction seen in only 35% of patients whose tonsils exceeded the palatal arches. The fact that airway narrowing represents a well-established factor for OSA development does not mean it serves as the sole factor. The development of sleep apnea in this patient population might be equally influenced by pharyngeal wall laxity and the dynamic tendency of the airway to collapse. Acromegaly-related soft tissue overgrowth continues to manifest as macroglossia, which studies show occurs in 84% of cases (22). Studies conducted through MRI have demonstrated that acromegalic patients exhibit greater tongue size measurements compared to healthy controls of a similar age group (7, 11). Medical treatment with somatostatin analogs results in a decrease of tongue size in patients (23). The measurements of tongue volume have demonstrated positive relationships with BMI and IGF-1 levels in patients who received treatment and those who had active acromegaly (22). We evaluated tongue position by using the FTP scale, which determines the relationship between the tongue and hard and soft palate. Our research revealed a connection between AHI values and FTP scores (p < 0.001), which demonstrates that both tongue hypertrophy and tongue placement in the oral cavity directly affect OSA presence in acromegaly patients. The analysis showed no meaningful relationship between FTP scores and BMI, which indicates that tongue growth in this population stems from disease factors rather than body weight.

To summarize, three out of four patients with newly diagnosed acromegaly had OSA which presented an additional cardiovascular risk. Our research demonstrated a high prevalence of OSA among patients with newly diagnosed acromegaly (76.67%), and that tongue base hypertrophy—as assessed by the FTP grading system—showed a statistically significant association with OSA severity (p < 0.001). On the other hand, we explicitly state that other anatomical parameters such as uvular thickness, tonsil size, and pharyngeal airway width did not demonstrate statistically significant associations with AHI values. The high prevalence of sleep-disordered breathing among this population provides sufficient justification for integrating OSA screening into standard initial diagnostic procedures. The early detection of sleep disorders enables healthcare providers to implement timely surgical and conservative treatments that focus on stopping obstructive sleep events.

The main limitation of this study is the relatively small sample size, which may have limited the statistical power of the analysis. Furthermore, due to the cross-sectional design, causal relationships cannot be inferred. Additionally, MRI data were incomplete for a few participants, which restricted full craniofacial evaluation. Future studies should include larger, multicenter cohorts and longitudinal follow-up to better assess the impact of treatment on craniofacial parameters and OSA severity in acromegaly.

## Data Availability

The original contributions presented in the study are included in the article/supplementary material. Further inquiries can be directed to the corresponding author.
